# Supplementary material for: Single-molecule imaging of microRNA-mediated gene silencing in cells
Source: Nat Commun. 2022 Mar 17;13:1435. doi: 10.1038/s41467-022-29046-5 (PMC8931058; doi:10.1038/s41467-022-29046-5)
Supplement: Supplementary file 1 — Supplementary Information [file 41467_2022_29046_MOESM1_ESM.pdf]

Kobayashi & Singer, Supplementary Fig. 1

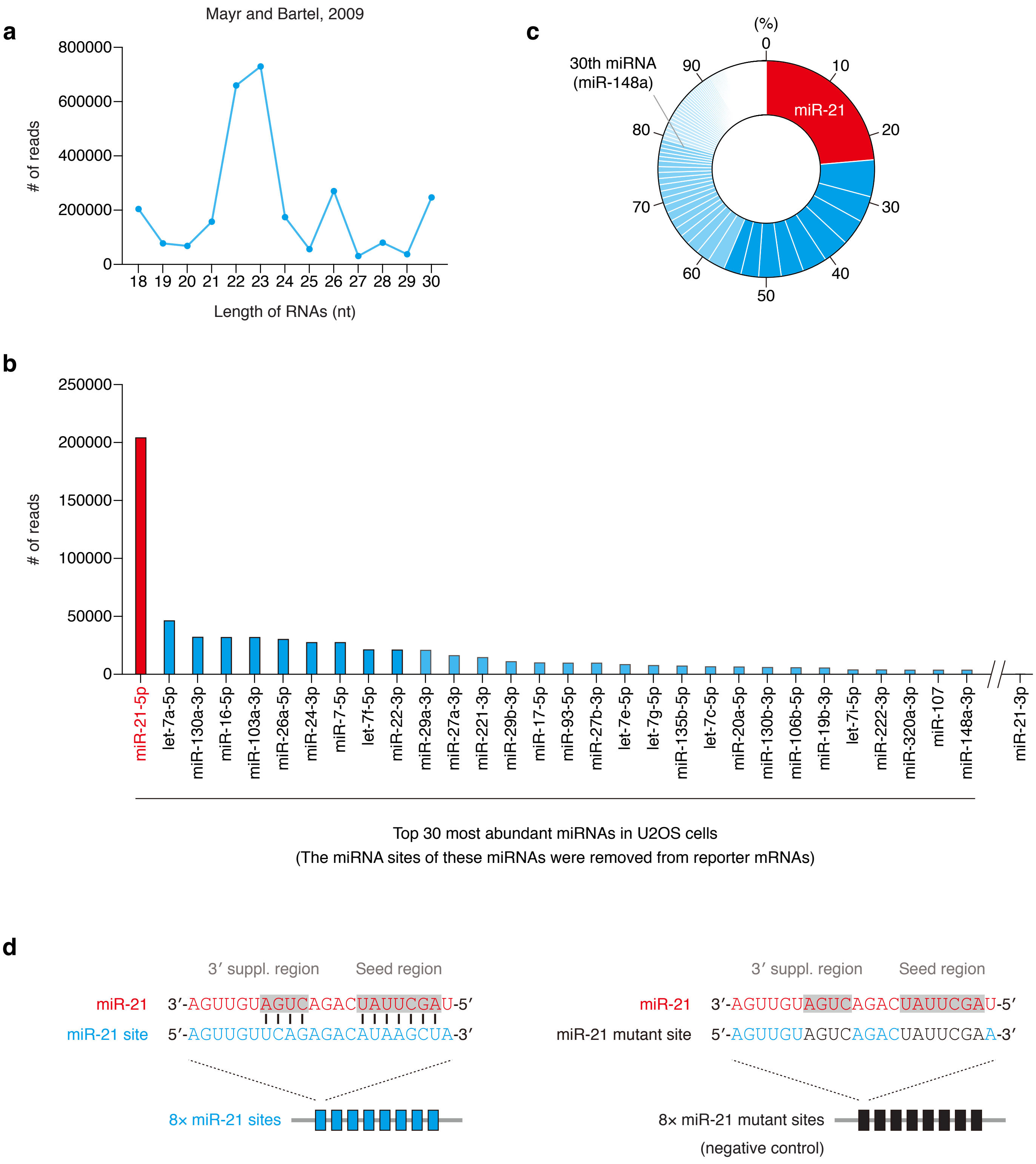

Supplementary Fig. 1. Expression profile of miRNAs in U2OS cells.

(**a**–**c**) The expression profile of miRNAs in U2OS cells. The small RNA-seq data (Mayr and Bartel, 2009) was reanalyzed. The length of small RNAs (a), the number of reads of each miRNA (b), and their relative occupancies (c) are shown. Top 30 most abundant miRNAs, whose sites were removed from reporter mRNAs, occupy ~80% of total population.

(**d**) The sequences of the miR-21 site (left) and the miR-21 mutant site (right). The miR-21 site was designed so that the positions 2–8 (seed region) and 13–16 (3′ supplemental region) of miR-21 form base-pairs with the miR-21 site. The miR-21 mutant site does not form base-pairs with miR-21. Source data are provided as a Source Data file.

1. Induction of transcription

▼

2. smFISH

Label *Fluc* mRNAs by smFISH probes (Quasar 570)

Label *SunTag* mRNAs by smFISH probes (Quasar 670)

Label nuclei by DAPI

▼

3. Image acquisition

3-color: *Fluc* mRNAs (orange), *SunTag* mRNAs (far red), nuclei (blue)

3D: pixel size: XY, 107.5 nm; Z, 200 nm

▼

4. Image analysis

Detect the outlines of cells and nuclei (by CellProfiler)

Detect spots of *Fluc* mRNAs and *SunTag* mRNAs (by FISH-quant)

Localize spots in 3D at sub-pixel resolution by fitting 3D Gaussians

Extract data of cytoplasmic spots: #, intensities, positions in X, Y, Z

▼

5. Data analysis

$$\text{Relative abundance of } \textit{SunTag} \text{ mRNAs} = \frac{\text{\# of } \textit{SunTag} \text{ mRNAs}}{\text{\# of } \textit{Fluc} \text{ mRNAs}}$$

Supplementary Fig. 2. Workflow for single-molecule imaging of miRNA-mediated mRNA decay.

The workflow for single-molecule imaging of miRNA-mediated mRNA decay is shown step by step. Detailed methods are provided in Methods.

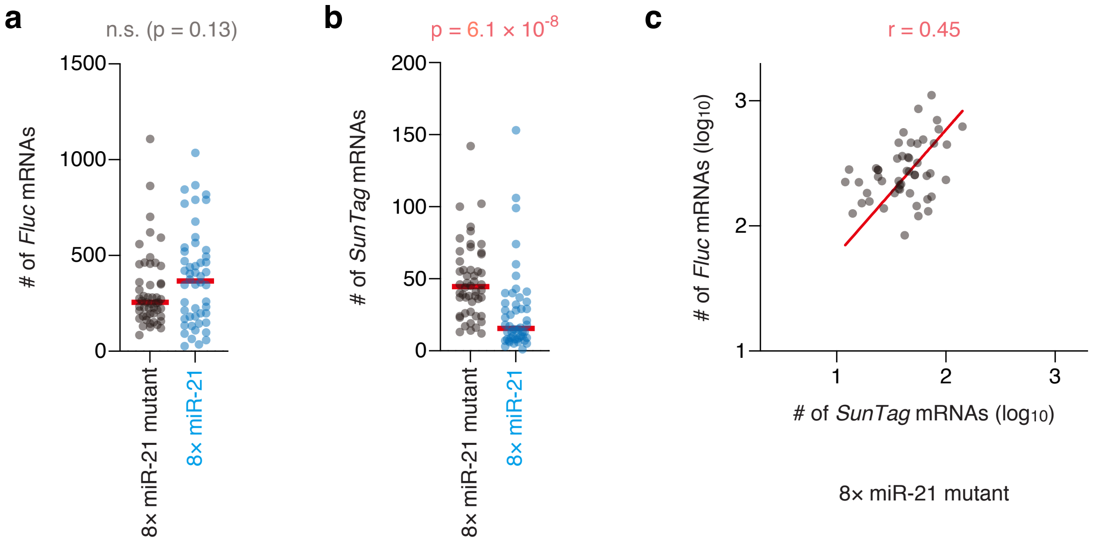
a
n.s. (p = 0.13)
1500
1000
500
0
# of Fluc mRNAs
8× miR-21 mutant
8× miR-21
b
p = 6.1 × 10-8
200
150
100
50
0
# of SunTag mRNAs
8× miR-21 mutant
8× miR-21
c
r = 0.45
3
2
1
# of Fluc mRNAs (log10)
1
2
3
# of SunTag mRNAs (log10)
8× miR-21 mutant

Supplementary Fig. 3. Supplemental data for Fig. 1.

(**a**, **b**) The number of *Fluc* mRNAs (a) and *SunTag* mRNAs (b) detected in U2OS cells. Images were analyzed using CellProfiler and FISH-quant. Each circle represents a single cell (n = 50 for each condition), while red lines represent the medians. The p values of one-tailed Mann Whitney test are shown. n.s., not significant.

(**c**) Positive correlation between the number of *Fluc* mRNAs and *SunTag* mRNAs. Each circle represents a single cell (n = 50), while the red line represents the result of simple linear regression. The Pearson correlation coefficient (r) is shown. Source data are provided as a Source Data file.

Kobayashi & Singer, Supplementary Fig. 4

1. Induction of transcription

2. SINAPS

Label SunTag peptides by anti-GCN4 antibodies (Alexa 488)

Label *SunTag* mRNAs by smFISH probes (Quasar 570)

Label nuclei by DAPI

3. Image acquisition

3-color: SunTag (green), mRNAs (orange), nuclei (blue)

3D: pixel size: XY, 107.5 nm; Z, 200 nm

4. Image analysis

Detect the outlines of cells and nuclei (by CellProfiler)

Detect spots of SunTag and mRNAs (by FISH-quant)

Localize spots in 3D at sub-pixel resolution by fitting 3D Gaussians

Extract data of cytoplasmic spots: #, intensities, positions in X, Y, Z

5. Colocalization analysis

Analyse colocalization based on the 3D distance between spots

Classify mRNAs into “untranslated” and “translated”

Classify SunTag into “free” and “on mRNAs”

6. Data analysis

$$\text{Translational efficiency} = \frac{\text{Intensity of SunTag on mRNAs}}{\text{\# of mRNAs}}$$

$$\text{Fraction of translated mRNAs} = \frac{\text{\# of translated mRNAs}}{\text{\# of mRNAs}}$$

$$\text{\# of ribosomes} = \frac{\text{Intensity of SunTag on mRNAs}}{\text{Intensity of free SunTag}}$$

Supplementary Fig. 4. Workflow for single-molecule imaging of miRNA-mediated translational repression.

The workflow for single-molecule imaging of miRNA-mediated translational repression is shown step by step. Detailed methods are provided in Methods.

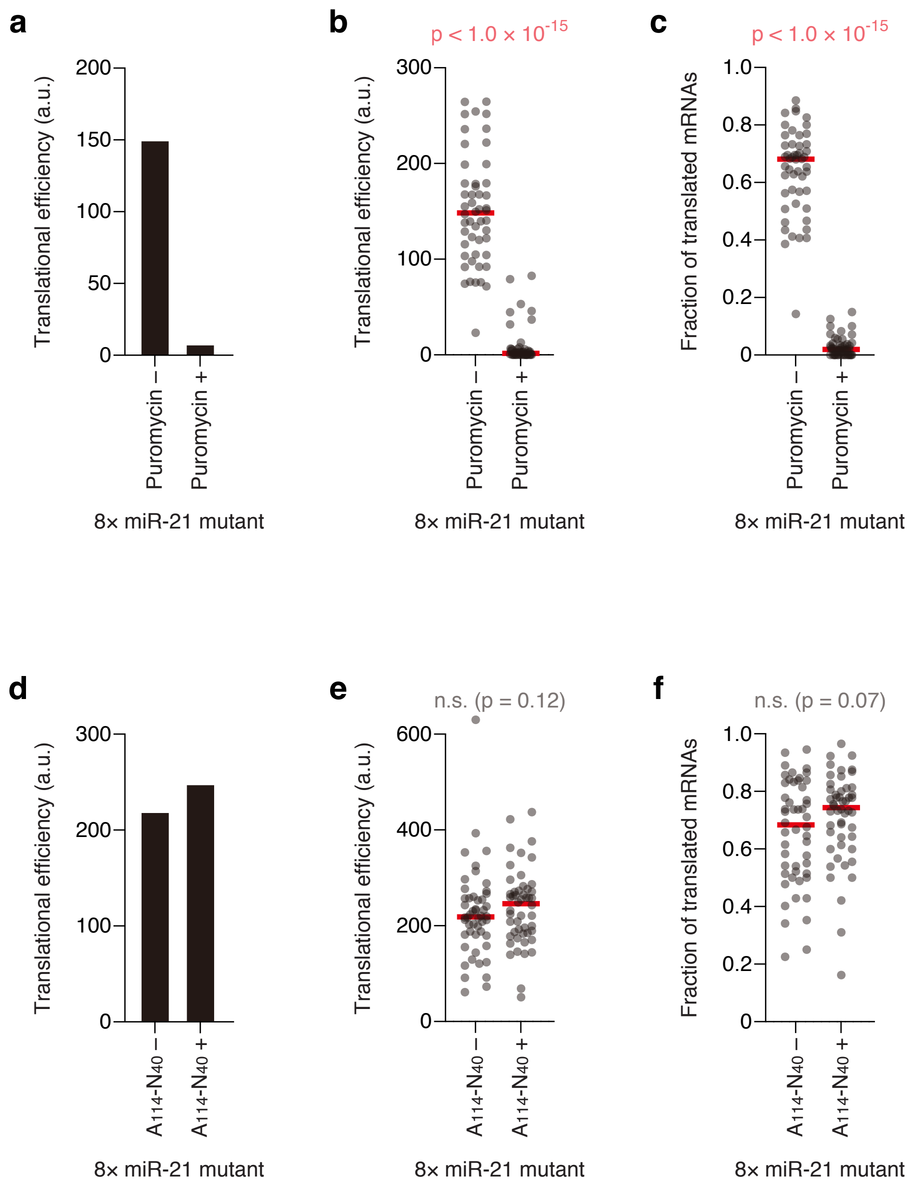
a
Translational efficiency (a.u.)
200
150
100
50
0
Puromycin –
Puromycin +
8× miR-21 mutant
b
p < 1.0 × 10-15
Translational efficiency (a.u.)
300
200
100
0
Puromycin –
Puromycin +
8× miR-21 mutant
c
p < 1.0 × 10-15
Fraction of translated mRNAs
1.0
0.8
0.6
0.4
0.2
0
Puromycin –
Puromycin +
8× miR-21 mutant
d
Translational efficiency (a.u.)
300
200
100
0
A114-N40 –
A114-N40 +
8× miR-21 mutant
e
n.s. (p = 0.12)
Translational efficiency (a.u.)
600
400
200
0
A114-N40 –
A114-N40 +
8× miR-21 mutant
f
n.s. (p = 0.07)
Fraction of translated mRNAs
1.0
0.8
0.6
0.4
0.2
0
A114-N40 –
A114-N40 +
8× miR-21 mutant

Supplementary Fig. 5. Validation of SINAPS experiments.

(**a**, **b**) Reduction of translational efficiency by puromycin treatment. Images were analyzed using CellProfiler and FISH-quant. Then, translational efficiency was calculated as described in Supplementary Fig. 4 (see also Methods). The results of bulk analysis (a) and single-cell analysis (b) are shown. In (b), each circle represents a single cell (n = 50 for each condition), while red lines represent the medians. The p value of one-tailed Mann Whitney test is shown. The puromycin – data in (a) and (b) are identical to the data in Fig. 2d and 2e, respectively.

(**c**) Reduction of the fraction of translated mRNAs by puromycin treatment. The fraction of translated mRNAs was calculated as described in Supplementary Fig. 4 (see also Methods). Each circle represents a single cell (n = 50 for each condition), while red lines represent the medians. The p value of one-tailed Mann Whitney test is shown. The puromycin – data are identical to the data in Fig. 2f.

(**d**, **e**) Translational efficiency of reporter mRNAs in the presence or absence of the A_{114}-N_{40} sequence. Images were analyzed using CellProfiler and FISH-quant. Then, translational efficiency was calculated as described in Supplementary Fig. 4 (see also Methods). The results of bulk analysis (d) and single-cell analysis (e) are shown. In (e), each circle represents a single cell (n = 50 for each condition), while red lines represent the medians. The p value of one-tailed Mann Whitney test is shown. n.s., not significant.

(**f**) The translated fraction of reporter mRNAs in the presence or absence of the A_{114}-N_{40} sequence. The fraction of translated mRNAs was calculated as described in Supplementary Fig. 4 (see also Methods). Each circle represents a single cell (n = 50 for each condition), while red lines represent the medians. The p value of one-tailed Mann Whitney test is shown. n.s., not significant. Source data are provided as a Source Data file.

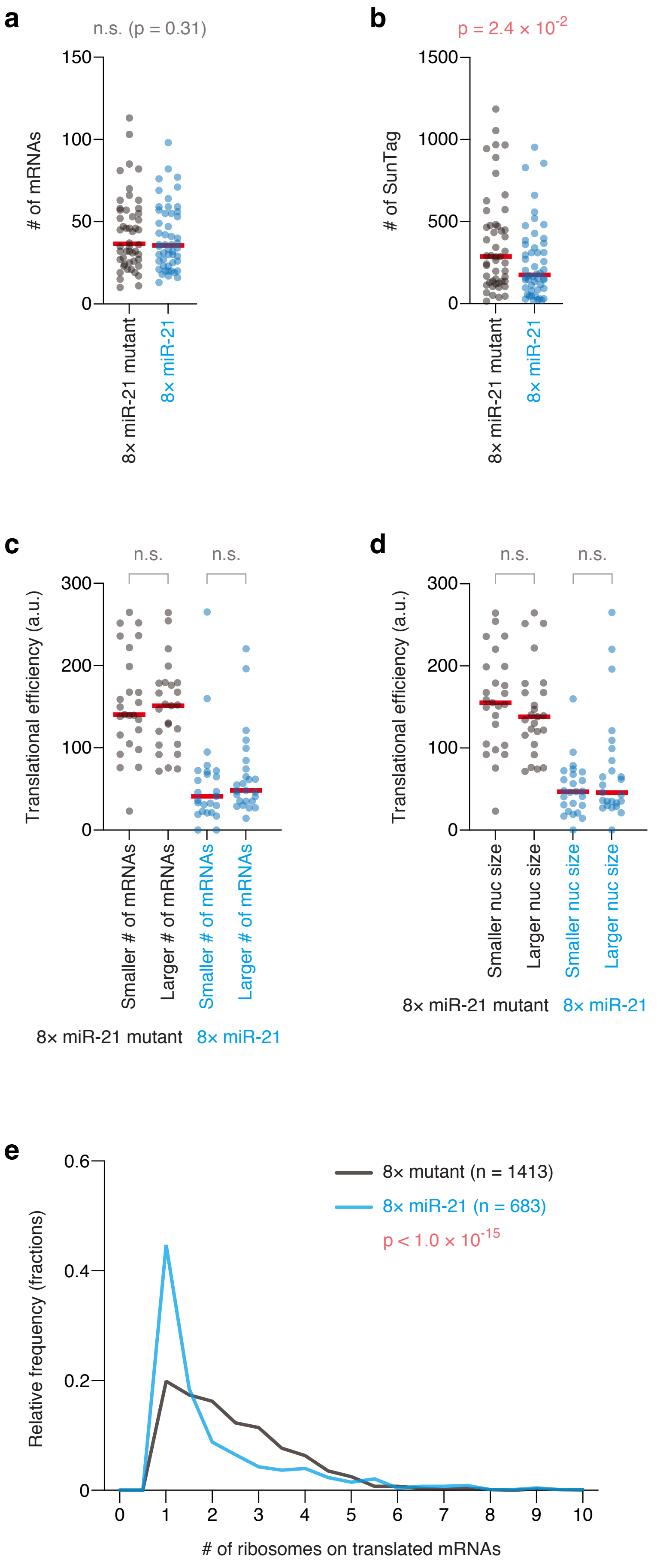
a
n.s. (p = 0.31)
# of mRNAs
150
100
50
0
8× miR-21 mutant
8× miR-21
b
p = 2.4 × 10-2
# of SunTag
1500
1000
500
0
8× miR-21 mutant
8× miR-21
c
n.s.
n.s.
Translational efficiency (a.u.)
300
200
100
0
Smaller # of mRNAs
Larger # of mRNAs
Smaller # of mRNAs
Larger # of mRNAs
8× miR-21 mutant
8× miR-21
d
n.s.
n.s.
Translational efficiency (a.u.)
Smaller nuc size
Larger nuc size
Smaller nuc size
Larger nuc size
8× miR-21 mutant
8× miR-21
e
Relative frequency (fractions)
0.6
0.4
0.2
0
8× mutant (n = 1413)
8× miR-21 (n = 683)
p < 1.0 × 10-15
0 1 2 3 4 5 6 7 8 9 10
# of ribosomes on translated mRNAs

Supplementary Fig. 6. Supplemental data for Fig. 2.

(**a**, **b**) The number of reporter mRNAs (a) and SunTag spots (b) detected in U2OS cells. Images were analyzed using CellProfiler and FISH-quant. Each circle represents a single cell (n = 50 for each condition), while red lines represent the medians. The p values of one-tailed Mann Whitney test are shown. n.s., not significant.

(**c**, **d**) Neither expression levels of mRNAs (c) nor nuclear sizes (d) affect translational efficiency of reporter mRNAs. The single-cell data in Fig. 2e were divided according to relative expression levels of mRNAs (c) or relative nuclear sizes (d). Each circle represents a single cell (n = 25 for each condition), while red lines represent the medians. The results of Dunn's multiple comparisons test are shown. n.s., not significant.

(**e**) Reduction of the number of ribosomes on translated mRNAs by miR-21. The number of ribosomes on translated mRNAs was calculated as described in Supplementary Fig. 4 (see also Methods). The p value of one-tailed Mann Whitney test is shown. Source data are provided as a Source Data file.

1. Induction of transcription

2. IF-FISH

Label *SunTag* mRNAs by smFISH probes (Quasar 570)

Label RISC by anti-AGO antibodies (Alexa 647)

Label nuclei by DAPI

3. Image acquisition

3-color: mRNAs (orange), RISC (far red), nuclei (blue)

3D: pixel size: XY, 107.5 nm; Z, 200 nm

4. Image analysis

Detect the outlines of cells and nuclei (by CellProfiler)

Detect spots of mRNAs and RISC (by FISH-quant)

Localize spots in 3D at sub-pixel resolution by fitting 3D Gaussians

Extract data of cytoplasmic spots: #, intensities, positions in X, Y, Z

5. Colocalization analysis

Analyse colocalization based on the 3D distance between spots

Classify mRNAs into “RISC-negative” and “RISC-positive”

Classify RISC into “free” and “on mRNAs”

6. Data analysis

$$\text{RISC-binding efficiency} = \frac{\text{Intensity of RISC on mRNAs}}{\text{\# of mRNAs}}$$

$$\text{Fraction of RISC-positive mRNAs} = \frac{\text{\# of RISC-positive mRNAs}}{\text{\# of mRNAs}}$$

Supplementary Fig. 7. Workflow for single-molecule imaging of RISC-binding.

The workflow for single-molecule imaging of RISC-binding is shown step by step. Detailed methods are provided in Methods.

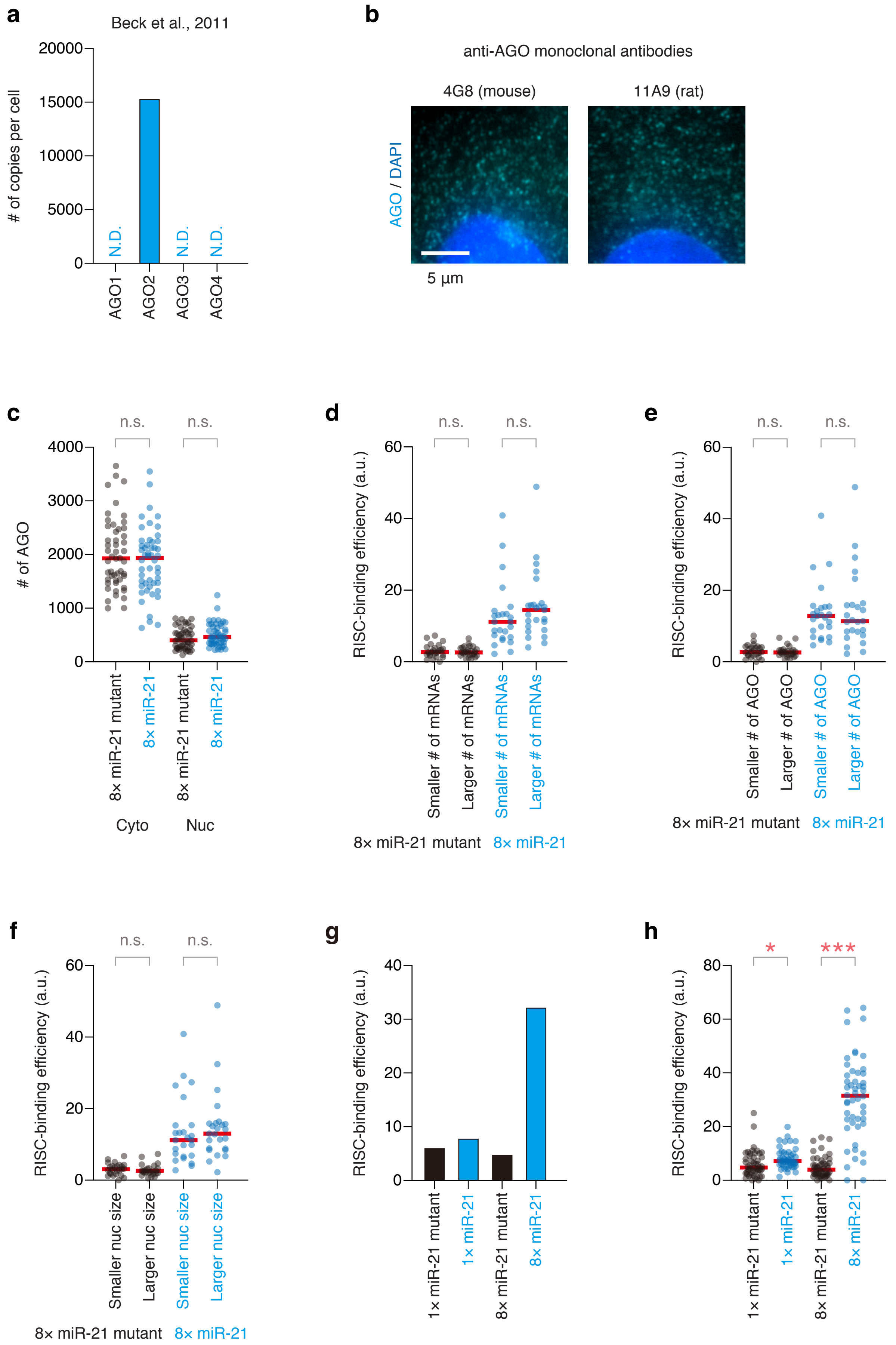
a
Beck et al., 2011
# of copies per cell
20000
15000
10000
5000
0
N.D.
N.D.
N.D.
AGO1
AGO2
AGO3
AGO4
b
anti-AGO monoclonal antibodies
4G8 (mouse)
11A9 (rat)
AGO / DAPI
5 µm
c
n.s.
n.s.
# of AGO
4000
3000
2000
1000
0
8× miR-21 mutant
8× miR-21
8× miR-21 mutant
8× miR-21
Cyto
Nuc
d
n.s.
n.s.
RISC-binding efficiency (a.u.)
60
40
20
0
Smaller # of mRNAs
Larger # of mRNAs
Smaller # of mRNAs
Larger # of mRNAs
8× miR-21 mutant
8× miR-21
e
n.s.
n.s.
RISC-binding efficiency (a.u.)
60
40
20
0
Smaller # of AGO
Larger # of AGO
Smaller # of AGO
Larger # of AGO
8× miR-21 mutant
8× miR-21
f
n.s.
n.s.
RISC-binding efficiency (a.u.)
60
40
20
0
Smaller nuc size
Larger nuc size
Smaller nuc size
Larger nuc size
8× miR-21 mutant
8× miR-21
g
RISC-binding efficiency (a.u.)
40
30
20
10
0
1× miR-21 mutant
1× miR-21
8× miR-21 mutant
8× miR-21
h
*
***
RISC-binding efficiency (a.u.)
80
60
40
20
0
1× miR-21 mutant
1× miR-21
8× miR-21 mutant
8× miR-21

Supplementary Fig. 8. Supplemental data for Fig. 3.

(**a**) The expression profile of AGO proteins in U2OS cells. The proteome data of U2OS cells (Beck et al., 2011) was reanalyzed. The number of copies per cell are shown. N.D., not detected. In U2OS cells, AGO2 is predominantly expressed.

(**b**) The images of U2OS cells immunostained with anti-AGO antibodies. Two distinct anti-AGO2 antibodies (the mouse monoclonal antibody, 4G8, and the rat monoclonal antibody, 11A9) showed similar IF patterns.

(**c**) The number of AGO spots detected in the cytoplasm (cyto) and in the nucleus (nuc). Images were analyzed using CellProfiler and FISH-quant. Each circle represents a single cell (n = 50 for each condition), while red lines represent the medians. The results of Dunn's multiple comparisons test are shown. n.s., not significant.

(**d**–**f**) Neither expression levels of mRNAs (d), expression levels of AGO (e), nor nuclear sizes (f) affect RISC-binding efficiency of reporter mRNAs. The single-cell data in Fig. 3d were divided according to relative expression levels of mRNAs (d), relative expression levels of AGO (e), or relative nuclear sizes (f). Each circle represents a single cell (n = 25 for each condition), while red lines represent the medians. The results of Dunn's multiple comparisons test are shown. n.s., not significant.

(**g**, **h**) RISC-binding to the 1× miR-21 reporter and the 8× miR-21 reporter. Images were analyzed using CellProfiler and FISH-quant. Then, RISC-binding efficiency was calculated as described in Supplementary Fig. 7 (see also Methods). The results of bulk analysis (g) and single-cell analysis (h) are shown. In (h), each circle represents a single cell (n = 50 for each condition), while red lines represent the medians. The results of Dunn's multiple comparisons test are shown. *** and * represent $p < 0.001$ and $p < 0.05$, respectively. p values were 2.8×10^{-2} (1× reporter) and $< 1.0 \times 10^{-15}$ (8× reporter). Source data are provided as a Source Data file.

1. Induction of transcription

2. SINAPS + IF-FISH

Label SunTag peptides by anti-GCN4 antibodies (Alexa 488)

Label *SunTag* mRNAs by smFISH probes (Quasar 570)

Label RISC by anti-AGO antibodies (Alexa 647)

Label nuclei by DAPI

3. Image acquisition

4-color: SunTag (green), mRNAs (orange), RISC (far red), nuclei (blue)

3D: pixel size: XY, 107.5 nm; Z, 200 nm

4. Image analysis

Detect the outlines of cells and nuclei (by CellProfiler)

Detect spots of SunTag, mRNAs, and RISC (by FISH-quant)

Localize spots in 3D at sub-pixel resolution by fitting 3D Gaussians

Extract data of cytoplasmic spots: #, intensities, positions in X, Y, Z

5. Colocalization analysis

Analyse colocalization based on the 3D distance between spots

Classify mRNAs into “untranslated” and “translated”

Classify SunTag into “free” and “on mRNAs”

Classify mRNAs into “RISC-negative” and “RISC-positive”

Classify RISC into “free” and “on mRNAs”

6. Data analysis

$$\text{Translational efficiency} = \frac{\text{Intensity of SunTag on mRNAs}}{\text{\# of mRNAs}}$$

$$\text{Fraction of translated mRNAs} = \frac{\text{\# of translated mRNAs}}{\text{\# of mRNAs}}$$

$$\text{\# of ribosomes} = \frac{\text{Intensity of SunTag on mRNAs}}{\text{Intensity of free SunTag}}$$

$$\text{RISC-binding efficiency} = \frac{\text{Intensity of RISC on mRNAs}}{\text{\# of mRNAs}}$$

$$\text{Fraction of RISC-positive mRNAs} = \frac{\text{\# of RISC-positive mRNAs}}{\text{\# of mRNAs}}$$

Supplementary Fig. 9. Workflow for simultaneous visualization of single mRNAs, translation, and RISC-binding.

The workflow for simultaneous visualization of single mRNAs, translation, and RISC-binding is shown step by step. Detailed methods are provided in Methods.

a

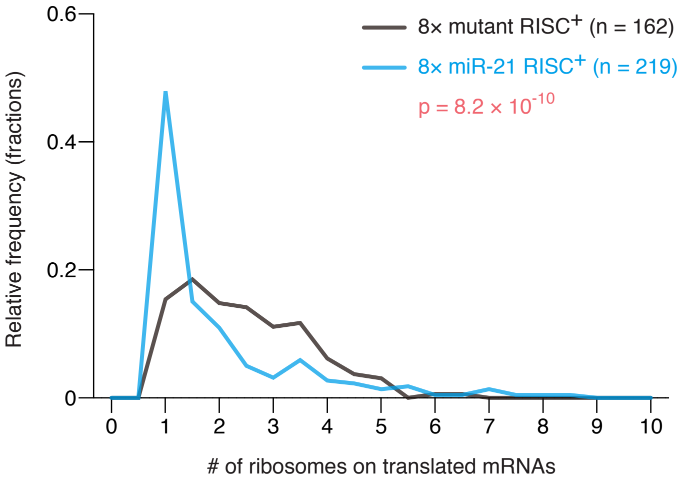
0.6
0.4
0.2
0
Relative frequency (fractions)
0
1
2
3
4
5
6
7
8
9
10
# of ribosomes on translated mRNAs
8× mutant RISC+ (n = 162)
8× miR-21 RISC+ (n = 219)
p = 8.2 × 10-10

b

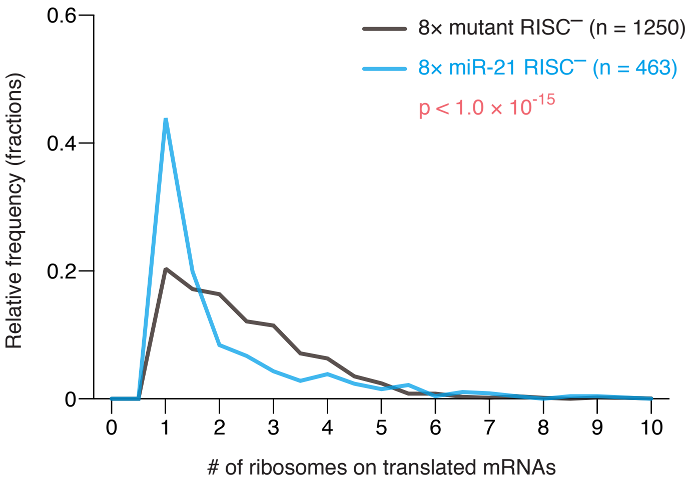
0.6
0.4
0.2
0
Relative frequency (fractions)
0
1
2
3
4
5
6
7
8
9
10
# of ribosomes on translated mRNAs
8× mutant RISC− (n = 1250)
8× miR-21 RISC− (n = 463)
p < 1.0 × 10-15

Supplementary Fig. 10. Supplemental data for Fig. 4.

(**a**, **b**) Reduction of the number of ribosomes on translated mRNAs by miR-21. RISC-positive mRNAs (a) and RISC-negative mRNAs (b) were selectively analyzed. The number of ribosomes on translated mRNAs was calculated as described in Supplementary Fig. 4 (see also Methods). The p values of one-tailed Mann Whitney test are shown. Source data are provided as a Source Data file.

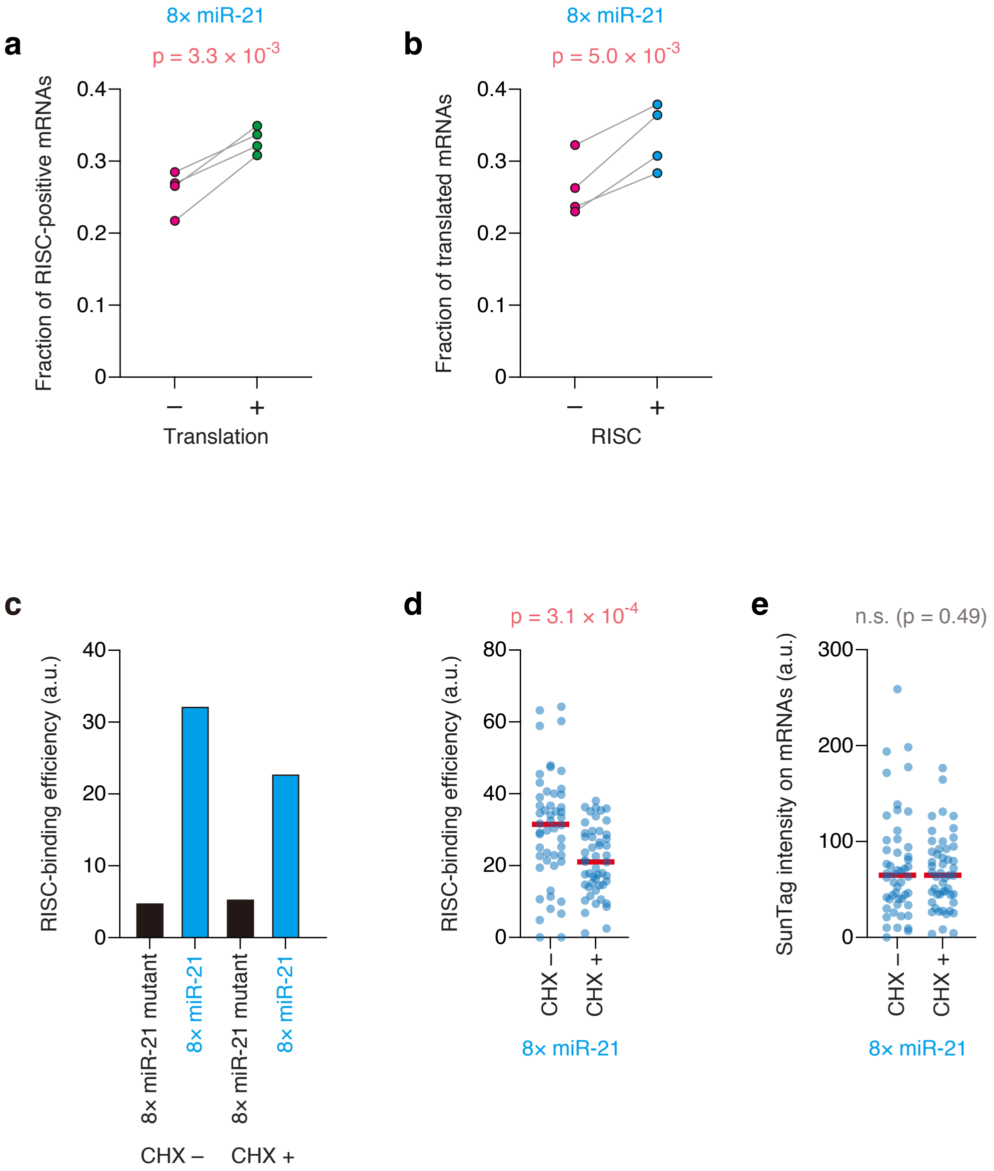
a
8× miR-21
p = 3.3 × 10-3
Fraction of RISC-positive mRNAs
0.4
0.3
0.2
0.1
0
−
+
Translation
b
8× miR-21
p = 5.0 × 10-3
Fraction of translated mRNAs
RISC
c
RISC-binding efficiency (a.u.)
40
30
20
10
8× miR-21 mutant
8× miR-21
CHX −
CHX +
d
p = 3.1 × 10-4
80
60
CHX −
CHX +
e
n.s. (p = 0.49)
SunTag intensity on mRNAs (a.u.)
300
200
100

Supplementary Fig. 11. RISC prefers translated mRNAs.

(**a**) Translated mRNAs tend to be RISC-positive mRNAs. The fraction of RISC-positive mRNAs and the p value of paired t test are shown (four biological replicates). Magenta and green circles represent the values of untranslated and translated mRNAs, respectively.

(**b**) RISC-positive mRNAs tend to be translated mRNAs. The fraction of translated mRNAs and the p value of paired t test are shown (four biological replicates). Magenta and cyan circles represent the values of RISC-negative and RISC-positive mRNAs, respectively.

(**c**, **d**) Reduction of RISC-binding efficiency by cycloheximide (CHX) treatment. Images were analyzed using CellProfiler and FISH-quant. Then, RISC-binding efficiency was calculated as described in Supplementary Fig. 7 (see also Methods). The results of bulk analysis (c) and single-cell analysis (d) are shown. In (d), each circle represents a single cell (n = 50 for each condition), while red lines represent the medians. The p value of one-tailed Mann Whitney test is shown. The CHX – data in (c) and (d) are identical to the data in Supplementary Fig. 8g and 8h, respectively.

(**e**) SunTag intensity on mRNAs in the presence or absence of CHX. Images were analyzed using CellProfiler and FISH-quant. Then, the total SunTag intensity on mRNAs was divided by the number of mRNAs. Each circle represents a single cell (n = 50 for each condition), while red lines represent the medians. The p value of one-tailed Mann Whitney test is shown. n.s., not significant. Source data are provided as a Source Data file.

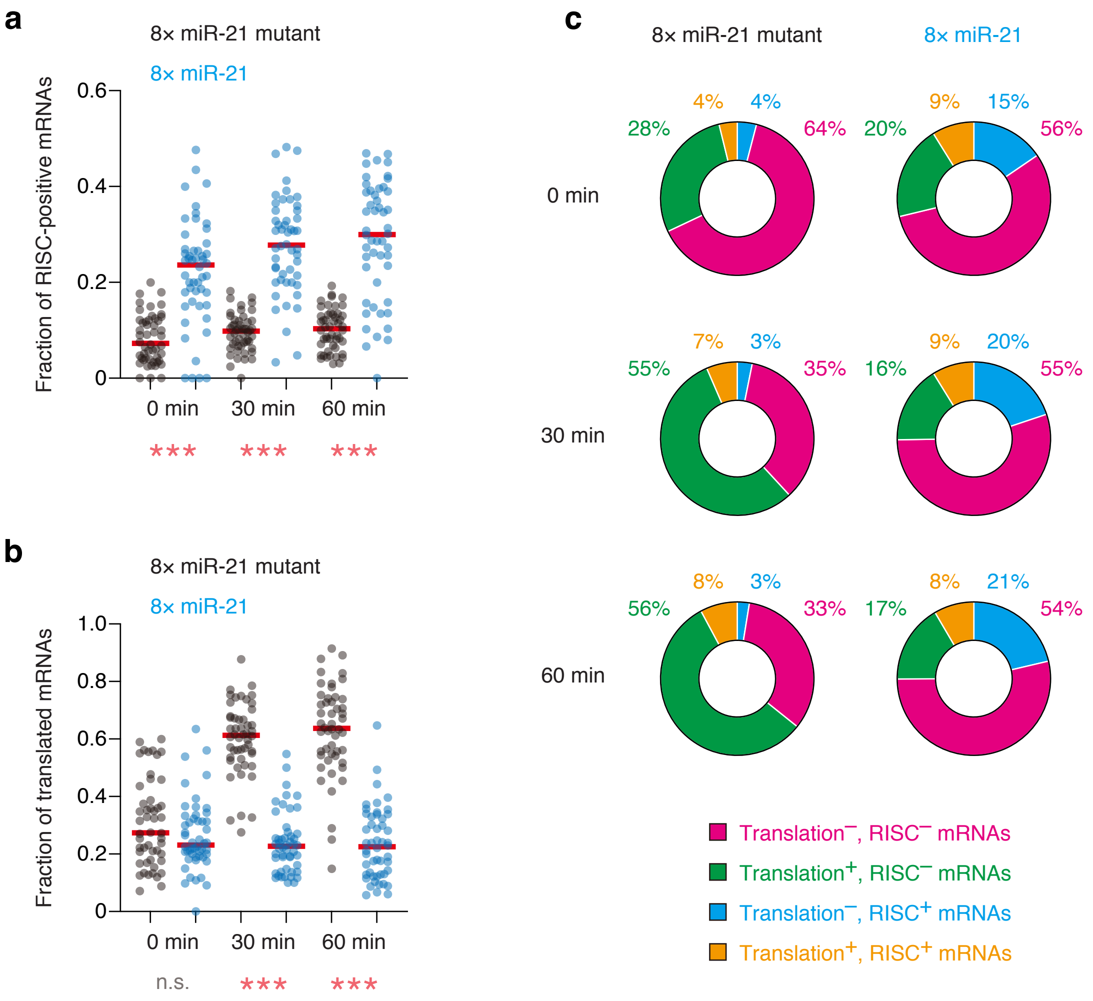
a
8× miR-21 mutant
8× miR-21
Fraction of RISC-positive mRNAs
0.6
0.4
0.2
0
0 min
30 min
60 min
***
***
***
b
8× miR-21 mutant
8× miR-21
Fraction of translated mRNAs
1.0
0.8
0.6
0.4
0.2
0
0 min
30 min
60 min
n.s.
***
***
c
8× miR-21 mutant
8× miR-21
0 min
28%
4%
4%
64%
20%
9%
15%
56%
30 min
55%
7%
3%
35%
16%
9%
20%
55%
60 min
56%
8%
3%
33%
17%
8%
21%
54%
Translation−, RISC− mRNAs
Translation+, RISC− mRNAs
Translation−, RISC+ mRNAs
Translation+, RISC+ mRNAs

Supplementary Fig. 12. Supplemental data for Fig. 5.

(**a**, **b**) Time-course analysis of RISC-binding (a) and translational repression (b) by single-mRNA imaging. Images were analyzed using CellProfiler and FISH-quant. Then, the fraction of RISC-positive mRNAs (a) and of translated mRNAs (b) were calculated as described in Supplementary Fig. 9 (see also Methods). Each circle represents a single cell (n = 50 for each condition), while red lines represent the medians. The results of Dunn's multiple comparisons test are shown. *** and n.s. represent $p < 0.001$ and not significant ($p > 0.05$), respectively. p values in (a) were 5.4×10^{-10} (0 min), 1.4×10^{-13} (30 min), and 3.2×10^{-12} (60 min), while those in (b) were 0.87 (0 min), $< 1.0 \times 10^{-15}$ (30 min), and $< 1.0 \times 10^{-15}$ (60 min).

(**c**) The ratio of RISC-negative untranslated (magenta), RISC-negative translated (green), RISC-positive untranslated (cyan), and RISC-positive translated (orange) mRNAs. All mRNAs were classified into these four classes based on 3D-colocalization analysis. Source data are provided as a Source Data file.

Supplementary Table 1. The sequences of smFISH probes used in this study.

smFISH probes toward *Fluc*

Sequence Name	Sequence
Fluc_1	catcggtgaaggcaatggtg
Fluc_2	taggtgatgtccacctcaat
Fluc_3	cgcacagacatctcgaagta
Fluc_4	tctcagagcacaccacgatg
Fluc_5	cactggcatgaagaactgca
Fluc_6	cactccgatgaacagggcac
Fluc_7	gtaaatgtcgttagcagggg
Fluc_8	cttagacacgaacaccacgg
Fluc_9	gtccatgatgatgatcttct
Fluc_10	cgaatgtgtacatgctctgg
Fluc_11	ctggcacgaagtcgtactcg
Fluc_12	gttttgtccctgtcgaaaga
Fluc_13	cagagctgttcatgatcagg
Fluc_14	cgtgagagaagcgcacacag
Fluc_15	tctggttgccgaaaataggg
Fluc_16	aatggcaccacgctcagaat
Fluc_17	agggtggtgaacatgccgaa
Fluc_18	aaagccgcaaatcaggtagc
Fluc_19	aagcggtacatcagcaccac
Fluc_20	agcagggcagactgaatttt
Fluc_21	gcgaagaagctgaacagggt
Fluc_22	cgtacttgtcgatcagggtg
Fluc_23	aatctcgtgcaggttagaca
Fluc_24	ctggcagatgaaagcgcttg
Fluc_25	taatcagaatggcgctggtt
Fluc_26	gaagaatggcaccaccttgc
Fluc_27	gacataatcatagggccgcg
Fluc_28	ctcagggttattcacgtagc
Fluc_29	cttgtcgatcagggcgtttg
Fluc_30	agtaggcaatgtcgccagag
Fluc_31	ccacgatgaagaagtgctcg
Fluc_32	ttgatcagagacttcaggcg
Fluc_33	aaatgttagggtgctgcagc
Fluc_34	acatagtccacgatctcctt
Fluc_35	ttcttagccttgatcaggat

smFISH probes toward *SunTag*

Sequence Name	Sequence
SunTag_1	ccacttcgttctcaagatga
SunTag_2	cccttttcagtctagctac
SunTag_3	aatttttgctcagcaactcc
SunTag_4	ttctttagtcgtgctacttc
SunTag_5	tttcgagagtaactcctcac
SunTag_6	ccacttcgttttcgagatga
SunTag_7	acttccctttttaagcgtg
SunTag_8	tcttggatagtagctcttca
SunTag_9	acctcgttctcaagatgata
SunTag_10	cggaacccttcttcaaacgc
SunTag_11	agttcttcgagagcagttcc
SunTag_12	gatccctttttaatcgagc
SunTag_13	tgaaagtagttcctcaccac
SunTag_14	cttcgttttcgaggtggtaa
SunTag_15	ccctgaacctttctttaatc
SunTag_16	tactcagtaattcttcaccc
SunTag_17	tttcgatagcaactcttcgc
SunTag_18	tttttgagcctagcaacttc
SunTag_19	ttttcgagagcaactcctcg
SunTag_20	acctcattttccaagtggta
SunTag_21	tttgctcaataactcctcgc
SunTag_22	cgcgacttcgttctctaaat
SunTag_23	ttcgataagagttcttcgcc
SunTag_24	ctcattttcgaggtggtagt
SunTag_25	agtggtagttcttgctcaag
SunTag_26	ttcaatctcgcgacctcatt
SunTag_27	attcttgctgagcaattcct
SunTag_28	cgacttcgttctccaaatga
SunTag_29	cgacttcattttccaagtgg
SunTag_30	ttgctcaataactcttcgcc
SunTag_31	ttcgttctccaagtggtaat
SunTag_32	agttcttcgataagagctcc
SunTag_33	gcgacttcattctctaagtg
SunTag_34	ttcttgctcaagagctcttc
SunTag_35	cacctcattttccaagtggt
SunTag_36	ttagatagtaactcttcccc
SunTag_37	cctcgttctcgagatgataa
SunTag_38	gatagttcttcgacaggagt
SunTag_39	ccttttaagtcttgcaacc
SunTag_40	ttactgagtagttcctcacc
SunTag_41	ttcgttttccaggtggtaat
SunTag_42	tcctgatcctttcttcaaac
SunTag_43	cttttgagagcagttcttcg
SunTag_44	gcaacctcattttccaaatg
SunTag_45	tgccacttccctttttaaa
SunTag_46	tttcgacagaagttcctcac
SunTag_47	gctacttcattctcgagatg
SunTag_48	gagccagaaccctttttaag

Supplementary Table 1. The sequences of smFISH probes used in this study.

The sequences of smFISH probes toward *Fluc* mRNAs and *SunTag* mRNAs used in this study are listed.
