# Supplementary material for: Single-molecule imaging of microRNA-mediated gene silencing in cells
Source: Nat Commun. 2022 Mar 17;13:1435. doi: 10.1038/s41467-022-29046-5 (PMC8931058; doi:10.1038/s41467-022-29046-5)
Supplement: Supplementary file 3 — Reporting Summary [file 41467_2022_29046_MOESM3_ESM.pdf]

Corresponding author(s): Hotaka Kobayashi & Robert H. Singer

Last updated by author(s): Feb 2, 2022

Reporting Summary

Nature Research wishes to improve the reproducibility of the work that we publish. This form provides structure for consistency and transparency in reporting. For further information on Nature Research policies, see our Editorial Policies and the Editorial Policy Checklist.

Statistics

For all statistical analyses, confirm that the following items are present in the figure legend, table legend, main text, or Methods section.

n/a	Confirmed	
☐	☒	The exact sample size (*n*) for each experimental group/condition, given as a discrete number and unit of measurement
☐	☒	A statement on whether measurements were taken from distinct samples or whether the same sample was measured repeatedly
☐	☒	The statistical test(s) used AND whether they are one- or two-sided<br>*Only common tests should be described solely by name; describe more complex techniques in the Methods section.*
☒	☐	A description of all covariates tested
☐	☒	A description of any assumptions or corrections, such as tests of normality and adjustment for multiple comparisons
☐	☒	A full description of the statistical parameters including central tendency (e.g. means) or other basic estimates (e.g. regression coefficient) AND variation (e.g. standard deviation) or associated estimates of uncertainty (e.g. confidence intervals)
☐	☒	For null hypothesis testing, the test statistic (e.g. F, t, r) with confidence intervals, effect sizes, degrees of freedom and P value noted<br>*Give P values as exact values whenever suitable.*
☒	☐	For Bayesian analysis, information on the choice of priors and Markov chain Monte Carlo settings
☒	☐	For hierarchical and complex designs, identification of the appropriate level for tests and full reporting of outcomes
☐	☒	Estimates of effect sizes (e.g. Cohen's *d*, Pearson's *r*), indicating how they were calculated

Our web collection on statistics for biologists contains articles on many of the points above.

Software and code

Policy information about availability of computer code

Data collection	Images were acquired using MetaMorph software (version 7.8.10.0, Molecular Devices), where the Multi Dimensional Acquisition mode was selected.
Data analysis	Images were analyzed using MATLAB R2018b Update 2 (9.5.0.1033004), FISH-quant v3 (Mueller et al., Nat Methods, 2013), CellProfiler 3.0 (McQuin et al., PLoS Biol, 2018), ImageJ software (Version: 2.1.0/1.53c), and GraphPad Prism (Version: 8 and 9).

For manuscripts utilizing custom algorithms or software that are central to the research but not yet described in published literature, software must be made available to editors and reviewers. We strongly encourage code deposition in a community repository (e.g. GitHub). See the Nature Research guidelines for submitting code & software for further information.

Data

Policy information about availability of data

All manuscripts must include a data availability statement. This statement should provide the following information, where applicable:

- Accession codes, unique identifiers, or web links for publicly available datasets
- A list of figures that have associated raw data
- A description of any restrictions on data availability

The small RNA-seq data analyzed in this study are available in the GEO database under accession code GSM416754 [https://www.ncbi.nlm.nih.gov/geo/query/acc.cgi?acc=GSM416754] (Mayr and Bartel, 2009). The proteome data used in this study are available at Beck et al., 2011 [https://www.embopress.org/doi/full/10.1038/msb.2011.82]. The imaging data generated in this study are available from the corresponding authors on reasonable request. Source data are provided with this paper.

Field-specific reporting

Please select the one below that is the best fit for your research. If you are not sure, read the appropriate sections before making your selection.

- [x] Life sciences
- [ ] Behavioural & social sciences
- [ ] Ecological, evolutionary & environmental sciences

For a reference copy of the document with all sections, see nature.com/documents/nr-reporting-summary-flat.pdf

Life sciences study design

All studies must disclose on these points even when the disclosure is negative.

Sample size	No sample-size calculation was performed. In pilot experiments, where we imaged and analyzed 20-30 cells, statistical significance between reporter mRNAs with and without miR-21 sites was confirmed. Thus, we determined that 50 cells are sufficient for our analyses.
Data exclusions	No data were excluded from the analyses.
Replication	All experiments were replicated at least two times. All attempts at replication were successful.
Randomization	Cells were randomly allocated into groups before nucleofection.
Blinding	Blinding was not done, because cells were assessed by the software described above in an unbiased manner.

Reporting for specific materials, systems and methods

We require information from authors about some types of materials, experimental systems and methods used in many studies. Here, indicate whether each material, system or method listed is relevant to your study. If you are not sure if a list item applies to your research, read the appropriate section before selecting a response.

Materials & experimental systems

n/a	Involved in the study	
☐	☒	Antibodies
☐	☒	Eukaryotic cell lines
☒	☐	Palaeontology and archaeology
☒	☐	Animals and other organisms
☒	☐	Human research participants
☒	☐	Clinical data
☒	☐	Dual use research of concern

Methods

n/a	Involved in the study	
☒	☐	ChIP-seq
☒	☐	Flow cytometry
☒	☐	MRI-based neuroimaging

Antibodies

Antibodies used	anti-GCN4 Rabbit antibody (Absolute Antibody, AB00436-23.0)<br>anti-AGO2 Mouse antibody (FUJIFILM Wako Pure Chemical, 015-22031, clone 4G8)<br>anti-AGO2 Rat antibody (MilliporeSigma, MABE253, clone 11A9)<br>anti-Rabbit IgG conjugated with Alexa Fluor 488 (Thermo Fisher, A-11034)<br>anti-Mouse IgG conjugated with Alexa Fluor 647 (Thermo Fisher, A-21236)
Validation	anti-GCN4 Rabbit antibody (https://absoluteantibody.com/product/anti-gcn4-c11l34/Ab00436-23.0_rabbit_igg/)<br>anti-AGO2 Mouse antibody (https://labchem-wako.fujifilm.com/us/product/detail/W01W0101-2203.html)<br>anti-AGO2 Rat antibody (https://www.sigmaaldrich.com/US/en/product/mm/mabe253)

Eukaryotic cell lines

Policy information about cell lines

Cell line source(s)	U2OS cells were obtained from ATCC.
Authentication	Genotyping through the Genomic Facility at the Albert Einstein College of Medicine.
Mycoplasma contamination	No mycoplasma contamination was detected.
Commonly misidentified lines (See ICLAC register)	No commonly misidentified cell lines were used.
